# Neonatal diagnosis of 49, XXXXY syndrome

**Published:** 2015-03

**Authors:** Katayoon Etemadi, Behnaz Basir, Safieh Ghahremani

**Affiliations:** 1 *Department of Molecular Medicine and Genetic, School of Medicine, Hamadan University of Medical Sciences, Hamadan, Iran.*; 2 *Department of Pediatrics, School of Medicine, Hamadan University of Medical Sciences, Hamadan, Iran.*; 3 *Cytogenetic Center, Shahid Beheshti Hospital, Hamadan University of Medical Sciences, Hamadan, Iran.*

**Keywords:** *49*, *XXXXY syndrome*, *Klinefelter syndrome*, *Intrauterine growth restriction*

## Abstract

**Background:**

49, XXXXY syndrome is a rare sex chromosomal disorder, occurring in 1 per 85,000-100,000 male births. The classical phenotype is ambiguous genitalia, facial dysmorphism, mental retardation and a combination of cardiac, skeletal and other malformations.

**Case:**

A two month-old boy with intrauterine growth restriction (IUGR) and low birth weight, facial dysmorphism, clinodactyly in feet, microphallus, and right undescendent testis were seen by neonatologist. Chromosomal studies via techniques of GTG-banding showed the constitution to be 49,XXXXY in all cells. He was visited by the pediatric cardiologist for congenital heart disease. No obvious malformation and congenital heart disease were seen.

**Conclusion:**

In the case, the main presentation of IUGR and low birth weight, clinodactyly with facial dysmorphism and genital abnormalities led to a suspicion of a sex chromosome aneuploidy which was subsequently confirmed by chromosomal analysis.

## Introduction

The 49,XXXXY syndrome was first reported in 1960 by Fraccaro *et al* and represents a rare sex chromosome aneuploidy syndrome with an approximate incidence of 1 in 85,000-100,000 male births (1). 49,XXXXY syndrome is known as a severe variant of Klinefelter syndrome due to its characteristic features, central nervous dysfunction, congenital anomalies, and global developmental delays (2). In affected boys clinically expressed as a combination of mental retardation, facial dysmorphism (ocular hypertelorism, upslanting palpebral fissures, and flat nasal bridge), genital abnormalities, cardiac, and skeletal malformations (3, 4).

More than one hundred cases with variable features have been reported in the world. Recently, two Iranian cases of 49,XXXXY syndrome were reported. The first is an eleven month old infant with congenital heart disease, facial malformations and ambiguous genitalia and the other was two Iranian cases: an infant and an adult in a family with 49,XXXXY syndrome (5, 6). In this study, we report the fourth Iranian case of 49, XXXXY syndrome based on intrauterine growth restriction (IUGR) and low birth weight, facial dysmorphism, clinodactyly in both feet, microphallus and right undescendent testis.

## Case Reports

**Figure 1:**
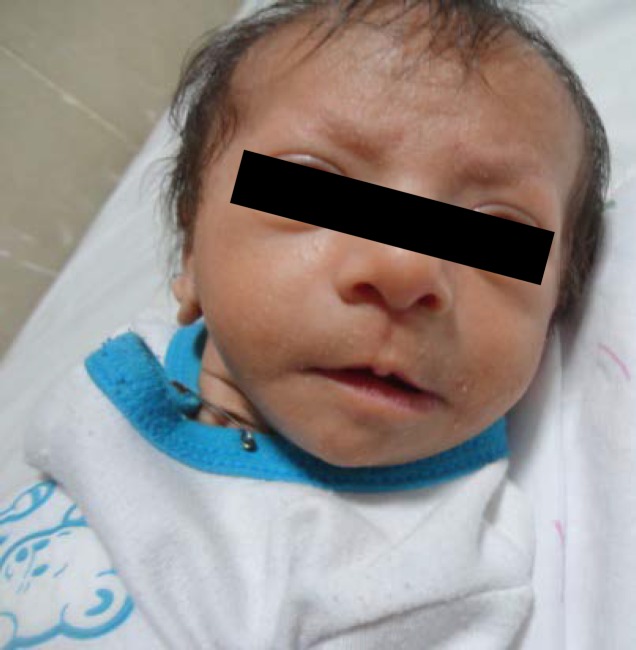
Facial dysmorphism, ocular hypertelorism in the patient

**Figure 2:**
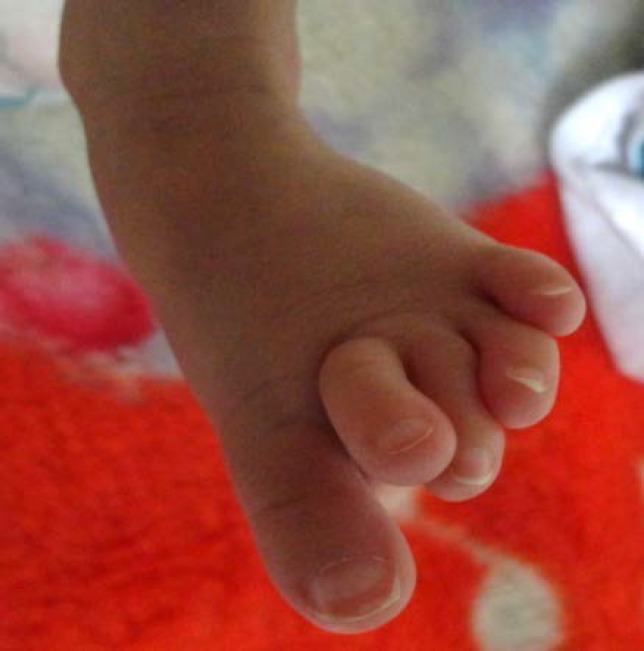
Bilateral feet finger clinodactyly

Our case is the fourth known case of Iranian origin of 49,XXXXY (Fraccaro) syndrome. He was the first child of family to a non-consanguineous, 26 years-old mother and 23 years-old father. He was born at 38 weeks of gestation by cesarean section (C/S) due to fetal distress and was admitted in Neonatal Intensive Care Unit (NICU) because of respiratory distress. The diagnosis was Transient Tachypnea of the Newborn (TTN) and he was discharged after 7 days with good respiratory condition. He had IUGR and low birth weight (2400 gr). The patient clinical examination revealed, facial dysmorphism, ocular hypertelorism and bilateral feet finger clinodactyly as shown in figures 1 and 2. One week after discharge, he was visited for poor feeding, low pitch cry and losing his weight (2200 gr). 

**Figure 3:**
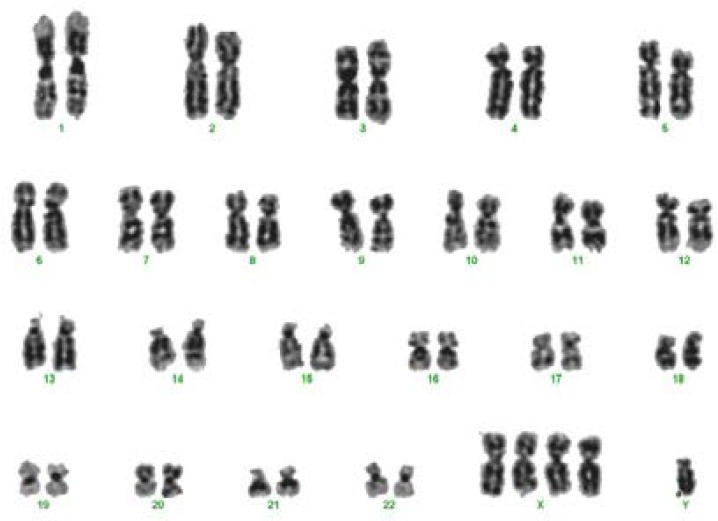
The karyogramm showing 49, XXXXY karyotype

He was examined by pediatric cardiologist for congenital heart disease. No obvious malformation and congenital heart disease were seen. He was received extra-care for weight gain. Based on these finding at the age of 2 months he referred to a cytogenetic clinic for chromosomal study. Chromosomal studies via techniques of GTG-banding showed the constitution to be 49,XXXXY in all cells (Figure 3). 

**Figure 4:**
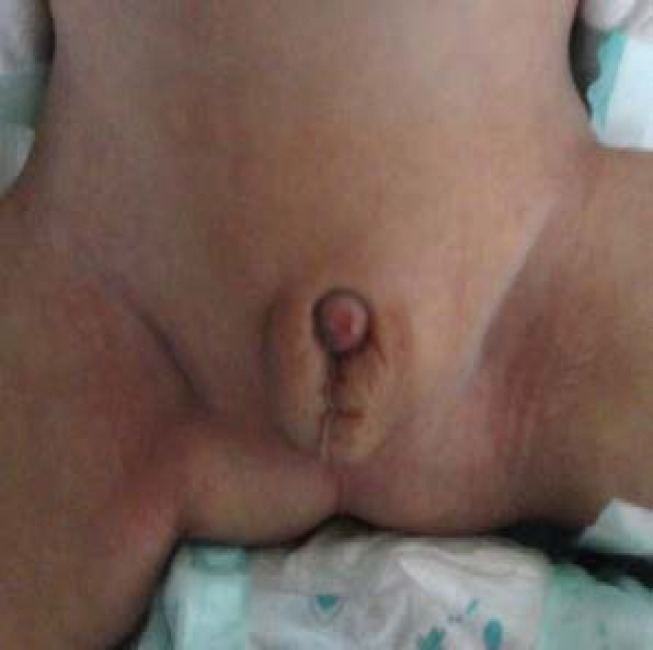
Genital abnormalities in the patient

The patient was follow up at the age of 9 months. He had 5.5 kg weight, 63 cm height and 40 cm head circumference, that total were <5^th^ percentile. He had developmental delay and hypotonia so he could not sit. Genital abnormalities comprise microphallus and right undescendent testis were seen in the patient (Figure 4).

## Discussion

Over 100 cases of 49,XXXXY syndrome have been published to date (7). Data on newborns with 49,XXXXY syndrome are still limited. The presence of multiple nonspecific congenital anomalies such as low birth weight, muscular hypotonic, clinodactyly, patent ductus arteriosus, scoliosis, or genital malformations has led to the diagnosis of 49,XXXXY syndrome in several newborns (8). The clinical phenotype changes as the person grows to an adult. Therefore, the certain features in the affected children are not necessarily present in adults and vice versa. “As with facial features, the body habitus of a subject with 49,XXXXY syndrome changes over his lifetime” (7). The parental origin and mechanism of formation of 49,XXXXY syndrome were studied in some cases. "A 49,XXXXY karyotype is thought to arise from maternal non-disjunction during both meiosis I and meiosis II. 

Such successive non-disjunction theoretically produces an egg with four X chromosomes, which, when fertilized by a Y bearing sperm, results in an embryo with 49,XXXXY syndrome. Interestingly, the occurrence of this syndrome does not appear to be related to maternal age. Two prevalent theories have been made to account for the phenotype associated with a 49,XXXXY genotype as well as for other X chromosome aneuploidies: 1) increased dosage of active genes in regions which escape X inactivation, and 2) asynchronous replication of the extra X chromosomes. Compared with Klinefelter syndrome (47,XXY), people with 49,XXXXY syndrome have characteristic facial features, particular habitus, cardiac defects, multiple skeletal anomalies, genital abnormalities and variable mental impairment. The number of the always active regions (at the tip of Xp) is increased from one to four, which cause the abnormal phenotype" (9). 

Many reports have paid attention to the distinctive phenotype of 49,XXXXY syndrome. In this case, the IUGR, weight loss, poor feeding, low pitch cry, clinodactyly and dysmorphic feature were the main characteristic which led to a suspicion of a sex chromosome aneuploidy that was confirmed by chromosomal analysis. At the 9^th^ month age genital abnormalities comprise microphallus and right undescendent testis were seen in the patient. No obvious malformation and congenital heart disease were seen. Early diagnosis offers multidisciplinary follow up involving pediatric sub-specialists as endocrinologists, cardiologists, and orthopedics. Moreover, parents can be counseled in detail and accompanied in the further clinical course.

"A subset of the clinical characteristics of patients with 48,XXXY and 49,XXXXY syndromes may be treated. For instance, early recognition and treatment of hyper gonadotropic hypogonadism seem to be crucial for adequate growth and pubertal development of patients with 49,XXXXY syndrome. Furthermore, there might be a potential benefit of early intervention with regard to neurodevelopment" (8).

## Conclusion

Prenatal diagnosis of the 49,XXXXY syndrome is generally fortuitous. In summary, we report on a case of neonatal diagnosis of 49,XXXXY syndrome based on IUGR, low birth weight, poor feeding, low pitch cry, dysmorphic feature, hypotonic, clinodactyly and genital abnormalities. It is suggested in similar cases to ensure no chromosomal abnormalities, karyotype study was initially attempted. At the end, it should be noted that parents of the case have expressed their consent to publish of the article. 

## Conflict of interest

We declare that we have no conflict of interest. An informed consent has been taken from parents of the patient to report this case.
